# Bioinformatic Analysis Reveals Bone Marrow Kinase as a Potential Diagnostic and Prognostic Biomarker for Multiple Cancer Types

**DOI:** 10.7759/cureus.68093

**Published:** 2024-08-29

**Authors:** Somia A Khalafallah, Ethar A Eltayeb Ahmed, Lubna S Elnour, Rihab Mohammed, Amna Makawi, Aml K Mohamed, Amna Balla, Marwa F. Alamin, Mohamed Alfaki

**Affiliations:** 1 Hematology and Immunohematology, Ibn-Sina University, Khartoum, SDN; 2 Hematology, Faculty of Health Sciences, National University of Malaysia, Kuala Lumpur, MYS; 3 Microbiology, Mycetoma Research Center, Khartoum, SDN; 4 Medical Microbiology, College of Medical Laboratory Sciences, University of Dongola, Al Dabbah, SDN; 5 Pediatrics, College of Medicine, Elrazi University, Khartoum, SDN; 6 Medicine and Surgery, International University of Africa, Khartoum, SDN; 7 Bioinformatics, University of Bahri, Khartoum, SDN; 8 Molecular Biology, Institute of Endemic Diseases, Khartoum University, Khartoum, SDN; 9 Research, Sidra Medicine, Doha, QAT

**Keywords:** bioinformatics, prognostic biomarker, diagnostic biomarker, cancer, bmx

## Abstract

Introduction: Bone marrow kinase, or BMX, is alternatively referred to as endothelial tyrosine kinase (Etk). It plays a vital role in the processes of cell proliferation, survival, immune activation, and the modulation of diverse signaling pathways. Since there are few direct comprehensive studies linking BMX role with multiple cancers, this study aimed to utilize bioinformatic tools to conduct a comprehensive analysis of BMX across multiple cancers, assessing its potential role.

Methods: Multiple databases including the Tumor Immune Estimation Resource (TIMER), Gene Expression Profiling Interactive Analysis (GEPIA), and University of Alabama at Birmingham Cancer Data Analysis Portal (UALCAN), have been used to explore BMX expression across different cancers, which has been further validated by using Gene Expression Omnibus (GEO) public datasets. In addition, we used the Kaplan-Meier plotter to estimate overall survival and cBioPortal for genetic alterations analysis. This study accommodates several other analyses like clinical parameters, immune cell infiltration, and DNA promoter methylation profiles to evaluate the general role of BMX in several cancers.

Results: The present investigation revealed that the BMX gene expression was significantly downregulated and could serve as an effective diagnostic biomarker in five types of cancers, namely breast invasive carcinoma (BRCA), colon adenocarcinoma (COAD), lung adenocarcinoma (LUAD), lung squamous cell carcinoma (LUSC), and rectum adenocarcinoma (READ) (all p < 0.05). Detailed analyses revealed notable downregulation of BMX in various clinical parameters such as age, gender, race, and cancer stage (all p < 0.05). To better understand the immunotherapeutic role of BMX, this investigation further examined the immune infiltration which exhibited positive correlations between BMX expression and the infiltration of immunological cells such as B cells, CD8+ T cells, CD4+ T cells, macrophages, neutrophils, and dendritic cells, especially in COAD, LUAD, and LUSC (all p < 0.05). In addition, the present study has demonstrated that diminished BMX gene expression is correlated with an unfavorable prognosis in kidney renal clear cell carcinoma (KIRC), liver hepatocellular carcinoma (LIHC), sarcoma (SARC), and uterine corpus endometrial carcinoma (UCEC); thus BMX gene expression can be used as a prognostic target for these specific cancers. Also, the results showed that the promoter methylation level of BMX was significantly elevated in LUAD and LUSC, whereas it was significantly decreased in BRCA (all p < 0.001). Importantly, our findings of significantly low BMX expression in LUAD and LUSC, along with their methylation profiles suggest that the low expression of BMX across these cancers is due to epigenetic factors. However, genetic alteration analysis revealed that mutations existed in only approximately 2% of the TCGA samples.

Conclusion: Our study revealed BMX as a diagnostic biomarker in BRCA, COAD, LUAD, LUSC, and READ and a prognostic biomarker in KIRC, LIHC, SARC, and UCEC. Furthermore, epigenetic variables may have a greater impact on BMX expression levels especially in LUAD and LUSC. This study also emphasized the role of BMX in the infiltration of immune cells, such as B cells, CD8+ T cells, CD4+ T cells, macrophages, neutrophils, and dendritic cells, in certain cancers. The BMX expression level highlights the prognostic value and potential therapeutic potential of BMX.

## Introduction

Cancer is a vast and diverse category of malignant tumor that results in high mortality rates globally [1]. On a global scale, cancer represents a significant public health challenge, thereby demanding the development of novel methodologies for early detection, accurate diagnosis, and improved patient prognoses. One of the most significant benefits of conquering this obstacle is the early discovery of biomarkers that function as indicators of the state of the disease. Considerable research has been conducted on cancer biomarkers, focusing on several categories, such as proteins, enzymes, nucleic acids, and circulating tumor cells (CTCs) [2-4].

Bone marrow kinase (BMX), also recognized as BMX nonreceptor tyrosine kinase or endothelial tyrosine kinase (Etk), as well as PSCTK2 and PSCTK3, is located on chromosome X. This particular gene encodes a nonreceptor tyrosine kinase that belongs to the Tec kinase family, a set of intracellular nonreceptor tyrosine kinases that includes five mammalian members, such as Bruton’s tyrosine kinase (Btk), tyrosine kinase (Tec), IL-2 inducible T-cell kinase (Itk), BMX, and tyrosine-protein kinase (Txk) [5]. Within the protein structure, there is a pleckstrin homology (PH) domain that is responsible for membrane directing through attaching to phosphatidylinositol 3,4,5-triphosphate (PIP3), and an Src homology, especially the SH2 domain that interacts with tyrosine-phosphorylated proteins to facilitate the transmission of signals. BMX is known to participate in various signal transduction pathways, like the Stat pathway, influencing the differentiation and carcinogenicity of different cancer cell types [6].

The predominant expression of BMX is observed in hematopoietic cells, whenever it engages in the immunological reaction; BMX executes a regulatory role in the release of proinflammatory cytokines triggered by TNFα, IL-1β, and TLR agonists in the context of the inflammatory reaction [7]. Its role is observed at the TAK1/TAB complex level and it is crucial for the initiation of the MAP kinase and NFκB signaling pathways. This protein not only participates in the response to ischemia and pressure overload in cardiac endothelium but has also been detected in various cancer types and very recently has been shown to mediate the survival and tumorigenicity of glioblastoma cancer stem cells [7]. Research has shown that BMX serves as a crucial mediator of proto-oncogene tyrosine-protein kinase, Src-induced cell transformation, and STAT3 activation, a vital pathway in cellular transformation [8]. Additionally, the blocking of BMX has been proven to boost the efficacy of chemotherapeutic agents by promoting BAK activation, causing tumor cells to become highly responsive to otherwise non-lethal doses of clinically important chemotherapeutic agents [1]. Our team decided to look into this interesting research topic because there has not been much direct research on the direct connection between the BMX gene and multiple cancers. This analysis aims to harness bioinformatics tools in conducting a comprehensive analysis of BMX across multiple cancers to assess its potential role as a diagnostic and prognostic biomarker.

## Materials and methods

TIMER database

Tumor Immune Estimation Resource (TIMER) is a comprehensive online platform that facilitates the thorough investigation of immunological infiltrates in diverse cancer types. It utilizes sophisticated algorithms to quantify the predominance of distinct immune cell populations according to patterns of gene expression. In this study, we applied the TIMER database (https://cistrome.shinyapps.io/timer/) [9] to assess the significance of BMX gene expression across various cancer types. Furthermore, TIMER was employed to determine the relationship between BMX gene expression and the quantity of distinct immune cells, illuminating the possibilities of immunological involvement of BMX in cancer.

GEPIA database

The Gene Expression Profiling Interactive Analysis (GEPIA) database offers an online platform for analyzing RNA sequencing expression data from the TCGA (The Cancer Genome Atlas) and GTEx (Genotype-Tissue Expression) databases [10]. It features customizable tools for differential expression analysis, profiling, graphing, and correlation analysis [10]. In this study, we employed the GEPIA database (http://gepia.cancer-pku.cn/index.html) to investigate BMX gene expression across diverse cancer types, corroborating the findings from the TIMER database. Additionally, we utilized GEPIA to assess the prognostic significance of BMX by generating Kaplan-Meier survival plots, which provided further insights into its potential influence on patient outcomes.

UALCAN database

The University of Alabama at Birmingham Cancer Data Analysis Portal (UALCAN) is a comprehensive and user-friendly digital platform that permits in-depth analysis of cancer-related omics data. It supports the identification and validation of potential biomarkers, generates visual representations of gene expression and patient survival trends, and facilitates the assessment of epigenetic regulation associated with genes of interest [11]. This study utilized the UALCAN database (https://ualcan.path.uab.edu/index.html) to assess BMX gene expression across a variety of cancer types, which validated the patterns of expression identified in the TIMER and GEPIA databases. Additionally, Kaplan-Meier survival analyses were conducted to explore the association between BMX expression and patient outcomes. Additionally, UALCAN assisted us in identifying connections between BMX expression and clinicopathological parameters such as age, gender, weight, cancer stage, and race. We also used UALCAN to investigate the epigenetic control of BMX expression through promoter methylation.

Kaplan-Meier plotter

The Kaplan-Meier plotter is a valuable online resource that enables the assessment of patient survival rates among a variety of tumor types. This database was employed to examine the correlation between the expression of all genes and survival outcomes, utilizing a comprehensive dataset encompassing more than 35,000 samples from 21 distinct cancer types [12]. The Kaplan-Meier plotter (available at https://kmplot.com/analysis/) was further utilized to evaluate the prognostic significance of BMX expression by generating Kaplan-Meier survival plots.

cBioPortal

The cBioPortal for Cancer Genomics is a valuable online platform that enables the interactive exploration of genetic alterations and multidimensional cancer genomics data [13]. In the present study, we utilized the cBioPortal (https://www.cbioportal.org/) to investigate the genetic changes associated with the BMX gene across various cancer types.

Validation of BMX expression

To validate our findings, we accessed publicly available datasets from the National Center for Biotechnology Information (https://www.ncbi.nlm.nih.gov). We conducted differential expression analysis via the GEO2R tool (https://www.ncbi.nlm.nih.gov/geo/geo2r), an interactive web-based application that enables researchers to compare multiple sample groups and identify differentially expressed genes (DEGs) [14]. This analysis validated the observed patterns of BMX gene expression across cancer types, corroborating the results from the databases mentioned previously. Differential expression profiles were then visualized using volcano plots generated through the SRplot platform (https://www.bioinformatics.com.cn/srplot), an online tool primarily utilized for data analysis and visualization of differentially expressed genes between cancer and normal tissues [15]. Statistical analyses were conducted based on the criteria of “|log2FC| > 1" and adjusted p-value < 0.05 to identify DEGs.

Statistical analysis

The Wilcoxon test was used in the TIMER database to compare the variation in gene expression levels between tumors and neighboring normal tissues, while the one-way analysis of variance (ANOVA) was used in the GEPIA database to compare expression and pathological stage. The Welch's T-test was used in all studies performed by the UALCAN database. For survival analysis, the log-rank test calculates the hazard ratio and p-value. The correlation of gene expression with the immune infiltration level was analyzed using partial Spearman’s correlation. What’s more, p < 0.05 was considered statistically significant.

## Results

BMX expression analysis across various cancers

**Figure 1:**
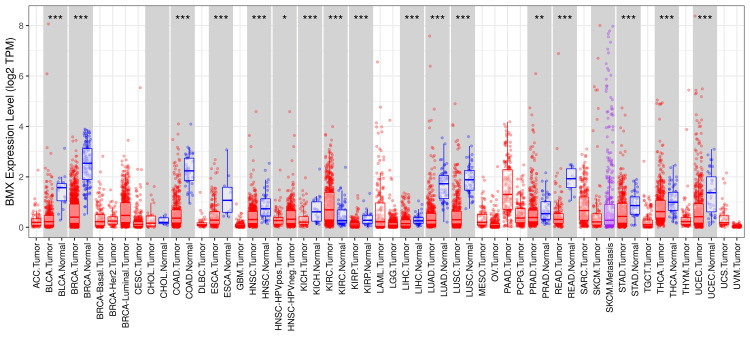
Expression of BMX in different cancer types according to the TIMER database. *p < 0.05, **p < 0.01, and ***p < 0.001. BMX: bone marrow kinase; TIMER: Tumor Immune Estimation Resource; p: p-value; ACC: adrenocortical carcinoma; BLCA: bladder urothelial carcinoma; BRCA: breast invasive carcinoma; Her2: human epidermal growth factor 2; CESC: cervical squamous cell carcinoma and endocervical adenocarcinoma; CHOL: cholangiocarcinoma; COAD: colon adenocarcinoma; DLBC: lymphoid neoplasm diffuse large B-cell lymphoma; ESCA: esophageal carcinoma; GBM: glioblastoma multiforme; HNSC: head and neck squamous cell carcinoma; HPVpos: human papillomavirus positive; HPVneg: human papillomavirus negative; KICH: kidney chromophobe; KIRC: kidney renal clear cell carcinoma; KIRP: kidney renal papillary cell carcinoma; LAML: acute myeloid leukemia; LGG: brain lower grade glioma; LIHC: liver hepatocellular carcinoma; LUAD: lung adenocarcinoma; LUSC: lung squamous cell carcinoma; MESO: mesothelioma; OV: ovarian serous cystadenocarcinoma; PAAD: pancreatic adenocarcinoma; PCPG: pheochromocytoma and paraganglioma; PRAD: prostate adenocarcinoma; READ: rectum adenocarcinoma; SARC: sarcoma; SKCM: skin cutaneous melanoma; STAD: stomach adenocarcinoma; TGCT: testicular germ cell tumors; THCA: thyroid carcinoma; THYM: thymoma; UCEC: uterine corpus endometrial carcinoma; UCS: uterine carcinosarcoma; UVM: uveal melanoma; TPM: transcript per million.

Three databases (TIMER, GEPIA, and UALCAN) were used to conduct a comprehensive analysis of BMX expression levels. First by starting the TIMER database which gives a general quick survey of the gene expression across multiple cancer types. TIMER database analysis revealed that the expression of BMX was significantly different from that in normal tissue in 16 cancer types. BMX was significantly downregulated in 15 cancer tissues, including bladder urothelial carcinoma (BLCA), breast invasive carcinoma (BRCA), colon adenocarcinoma (COAD), esophageal carcinoma (ESCA), head and neck squamous cell carcinoma (HNSC), kidney chromophobe (KICH), kidney renal papillary cell carcinoma (KIRP), liver hepatocellular carcinoma (LIHC), lung adenocarcinoma (LUAD), lung squamous cell carcinoma (LUSC), prostate adenocarcinoma (PRAD), rectum adenocarcinoma (READ), stomach adenocarcinoma (STAD), thyroid carcinoma (THCA) and uterine corpus endometrial carcinoma (UCEC). Moreover, BMX expression was significantly upregulated in kidney renal clear cell carcinoma (KIRC). We also found that the expression of BMX in head and neck squamous cell carcinoma (HNSC) patients who had human papillomavirus (HPV) infection was significantly lower than that in HNSC patients without HPV infection (Figure 1).

**Figure 2:**
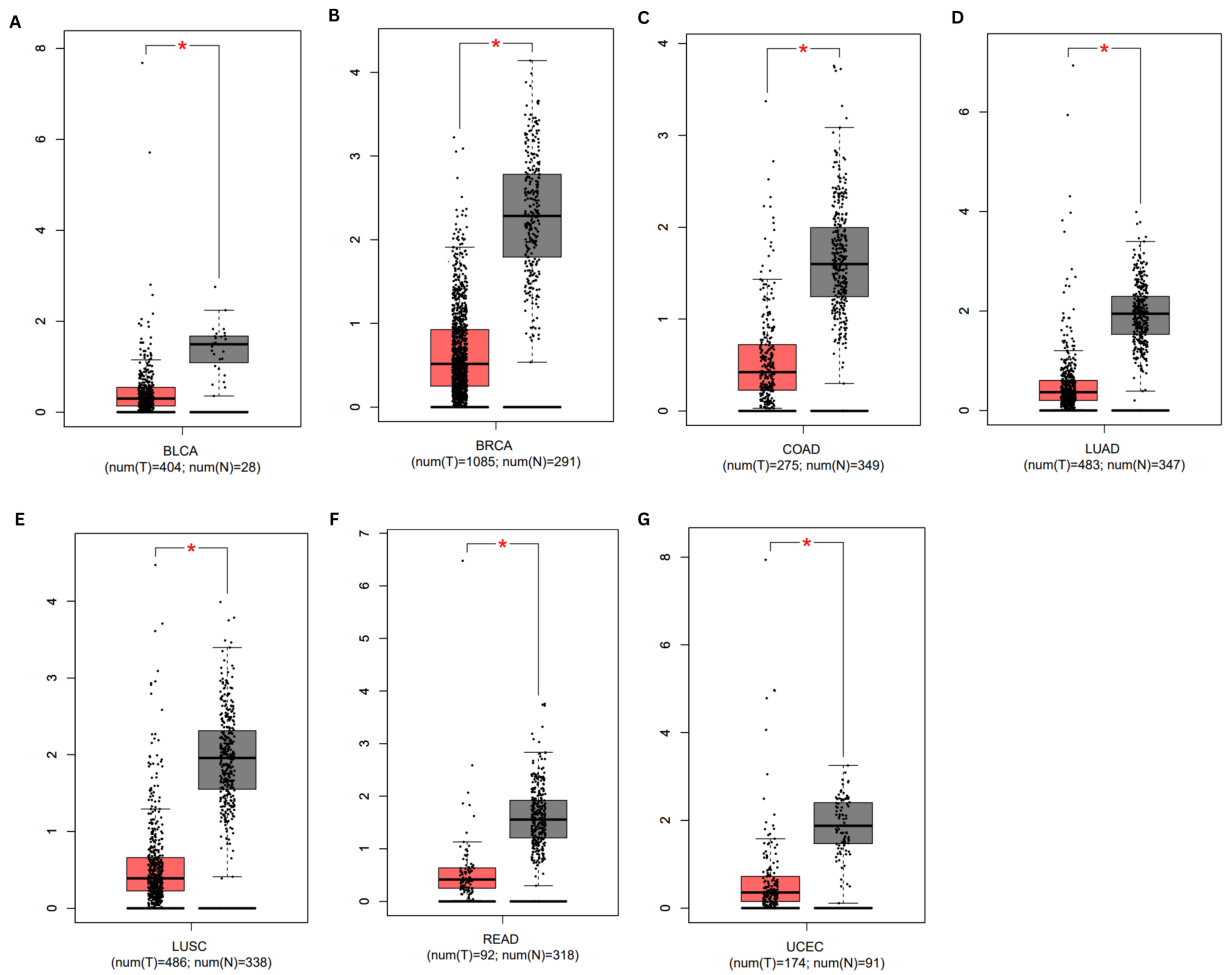
Expression of BMX in different cancer types according to the GEPIA database. (A) BMX expression in BLCA. (B) BMX expression in BRCA. (C) BMX expression in COAD. (D) BMX expression in LUAD. (E) BMX expression in LUSC. (F) BMX expression in READ. (G) BMX expression in UCEC. *p < 0.05. BMX: bone marrow kinase; GEPIA: Gene Expression Profiling Interactive Analysis; p: p-value; BLCA: bladder urothelial carcinoma; BRCA: breast invasive carcinoma; COAD: colon adenocarcinoma; LUAD: lung adenocarcinoma; LUSC: lung squamous cell carcinoma; READ: rectum adenocarcinoma; UCEC: uterine corpus endometrial carcinoma; num(T): the number of tumor samples; num(N): the number of normal samples; red color: tumor samples; gray color: normal samples; Y axis: level of BMX expression in transcripts per million.

Second, using the GEPIA database, we further examined the expression of BMX in 16 different malignancies retrieved from TIMER, we found that BMX was consistently significantly downregulated in BLCA, BRCA, COAD, LUAD, LUSC, READ, and UCEC tissues compared with normal tissues (Figure 2). In contrast, there was no significant difference in BMX expression between ESCA, HNSC, KICH, KIRC, KIRP, LIHC, PRAD, STAD, and THCA tissues and normal tissue (Appendix A).

**Figure 3:**
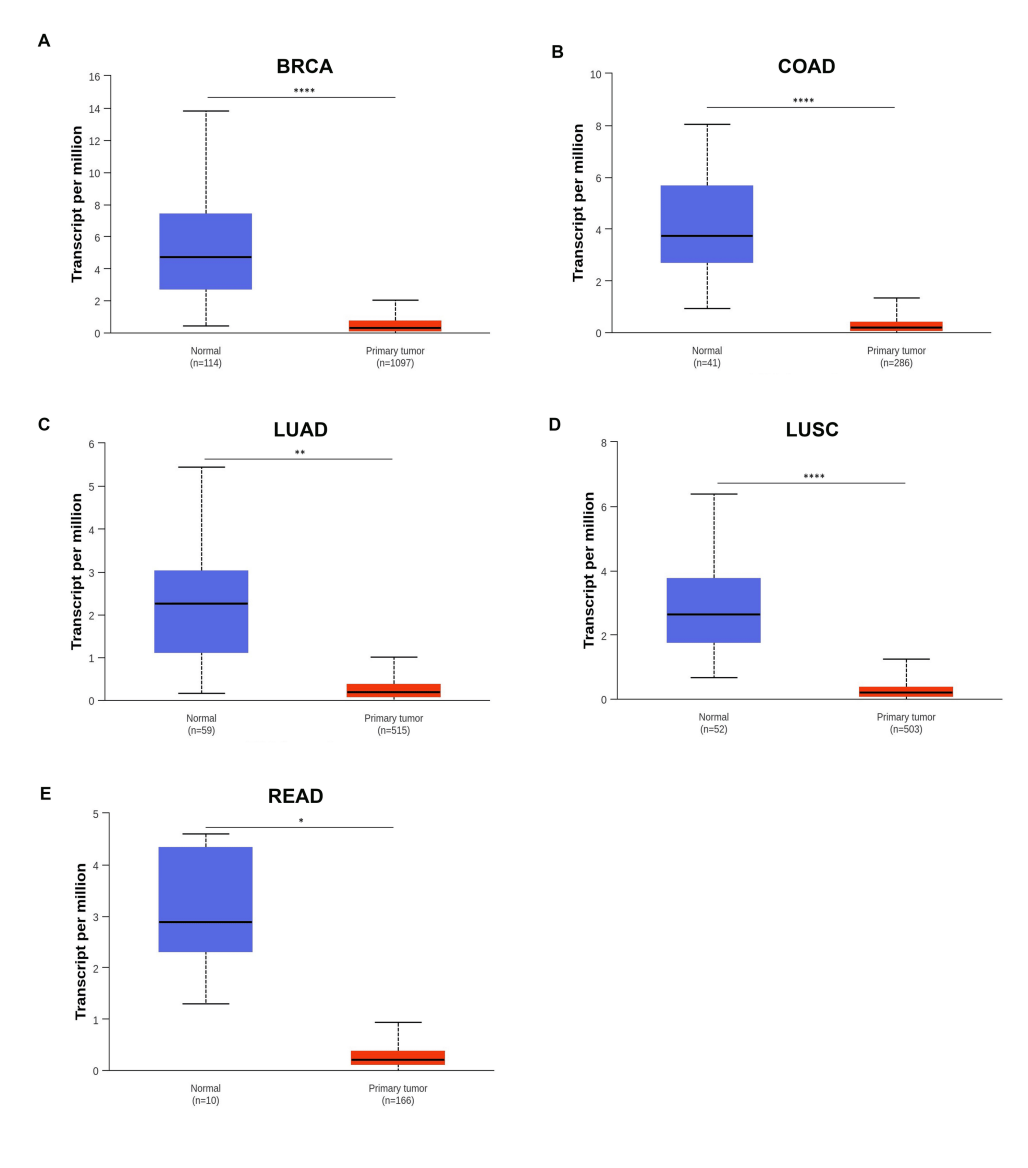
BMX expression in different cancer types from the UACLAN database. (A) BMX expression in BRCA. (B) BMX expression in COAD. (C) BMX expression in LUAD. (D) BMX expression in LUSC. (E) BMX expression in READ. *p < 0.05, **p < 0.01, and ****p < 0.0001. BMX: bone marrow kinase; UALCAN: University of Alabama at Birmingham Cancer Data Analysis Portal; p: p-value; BRCA: breast invasive carcinoma; COAD: colon adenocarcinoma; LUAD: lung adenocarcinoma; LUSC: lung squamous cell carcinoma; READ: rectum adenocarcinoma.

Finally, we verified that BMX gene expression in seven cancer samples was consistent according to the TIMER and GEPIA databases using the UACLAN database. BMX expression was significantly downregulated in BRCA, COAD, LUAD, LUSC, and READ tissues compared to the normal tissues (Figure 3). On the other hand, there was no significant difference in BMX expression between BLCA and UCEC tissues and normal tissue (Appendix B). The consistent downregulation of BMX observed across the three datasets in BRCA, COAD, LUAD, LUSC, and READ suggests that BMX may have potential as a diagnostic biomarker for these cancer types. We focused our additional investigations on these five cancer types.

BMX expression analysis according to certain clinical parameters

According to TIMER, GEPIA, and UACLAN; five cancers (BRCA, COAD, LUAD, LUSC, and READ) consistently presented significantly lower levels of BMX expression than normal tissues. In these tumors, the expression of the BMX gene was also thoroughly examined with respect to specific clinical parameters. Patients were classified by age, gender, race, and weight. The age groups were as follows: young adults (21-40 years), middle-aged adults (41-60 years), older adults (61-80 years), and elderly (81-100 years). The gender groups included male and female patients and the race groups comprised Caucasian, African American, and Asian patients. The weight groups included normal weight, extreme weight, obese, and extremely obese.

**Figure 4:**
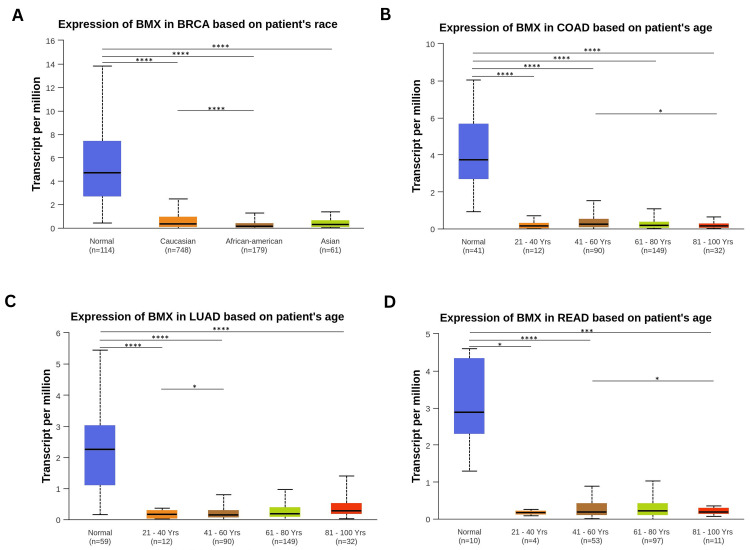
BMX expression analysis in cancer patients using the UALCAN database based on specific clinical parameters. (A) BMX expression in BRCA patients according to race. (B) Expression of BMX in COAD patients based on age. (C) BMX expression in LUAD patients according to age. (D) BMX expression in READ patients based on age. *p < 0.05, ***p < 0.001, and ****p<0.0001. BMX: bone marrow kinase; UALCAN: University of Alabama at Birmingham Cancer Data Analysis Portal; p: p-value; BRCA: breast invasive carcinoma; COAD: colon adenocarcinoma; LUAD: lung adenocarcinoma; READ: rectum adenocarcinoma.

The study found that BMX expression was markedly reduced across all age categories, genders, and racial groups in BRCA and COAD patient cohorts, compared to normal populations (all p < 0.0001; Appendix C). Specifically, African American BRCA patients exhibited significantly lower BMX expression levels than their Caucasian counterparts (p < 0.0001; Figure 4A). While middle-aged adults (41-60 years) and elderly (81-100 years) COAD patients differed significantly in BMX expression (p < 0.05; Figure 4B). For LUAD, BMX was significantly downregulated in young adults (21-40 years), middle-aged adults (41-60 years), and older adults (81-100 years) (all p < 0.0001), with middle-aged adults (41-60 years) differing significantly from young adults (21-40 years) (p < 0.05), while older adults (61-80 years) showed no significant difference from the normal population (Figure 4C).

Males exhibited significant downregulation of BMX (p < 0.0001) while females did not, and only Caucasian and African American patients showed significant downregulation among racial groups (p < 0.05) compared to the normal population (Appendix C). LUSC patients exhibited significant downregulation in middle-aged adults (41-60 years), older adults (61-80 years), and elderly (81-100 years) patients (all p < 0.0001), while data for young adults (21-40 years) were unavailable due to the small sample size, which was insufficient for statistical analysis; both genders and all races showed significant downregulation compared to the normal population (all p < 0.0001; Appendix C). In READ patients, BMX was significantly downregulated in young adults (21-40 years), middle-aged adults (41-60 years), and elderly (81-100 years) patients compared to the normal population (all p < 0.05), while there was no significant difference in its expression between older adults (61-80 years) and the normal group (p > 0.05). Furthermore, there was a significant difference between middle-aged adults (41-60 years) and elderly (81-100 years) patients (p < 0.05; Figure 4D). Compared with the normal population, females exhibited significant downregulation of BMX (p < 0.001), whereas males did not, and only African American patients showed significant downregulation among racial groups (p < 0.0001) (Appendix C). Additionally, in both COAD and READ patients, all weight groups were significantly downregulated compared to those in the normal population (all p < 0.0001; Appendix C), while no weight data were available for BRCA, LUAD, or LUSC in the database. In conclusion, the BMX expression levels were consistently downregulated in all clinical parameters with differences in age groups across the COAD, LUAD, and READ patients, in addition to differences in BRCA according to the race groups.

**Figure 5:**
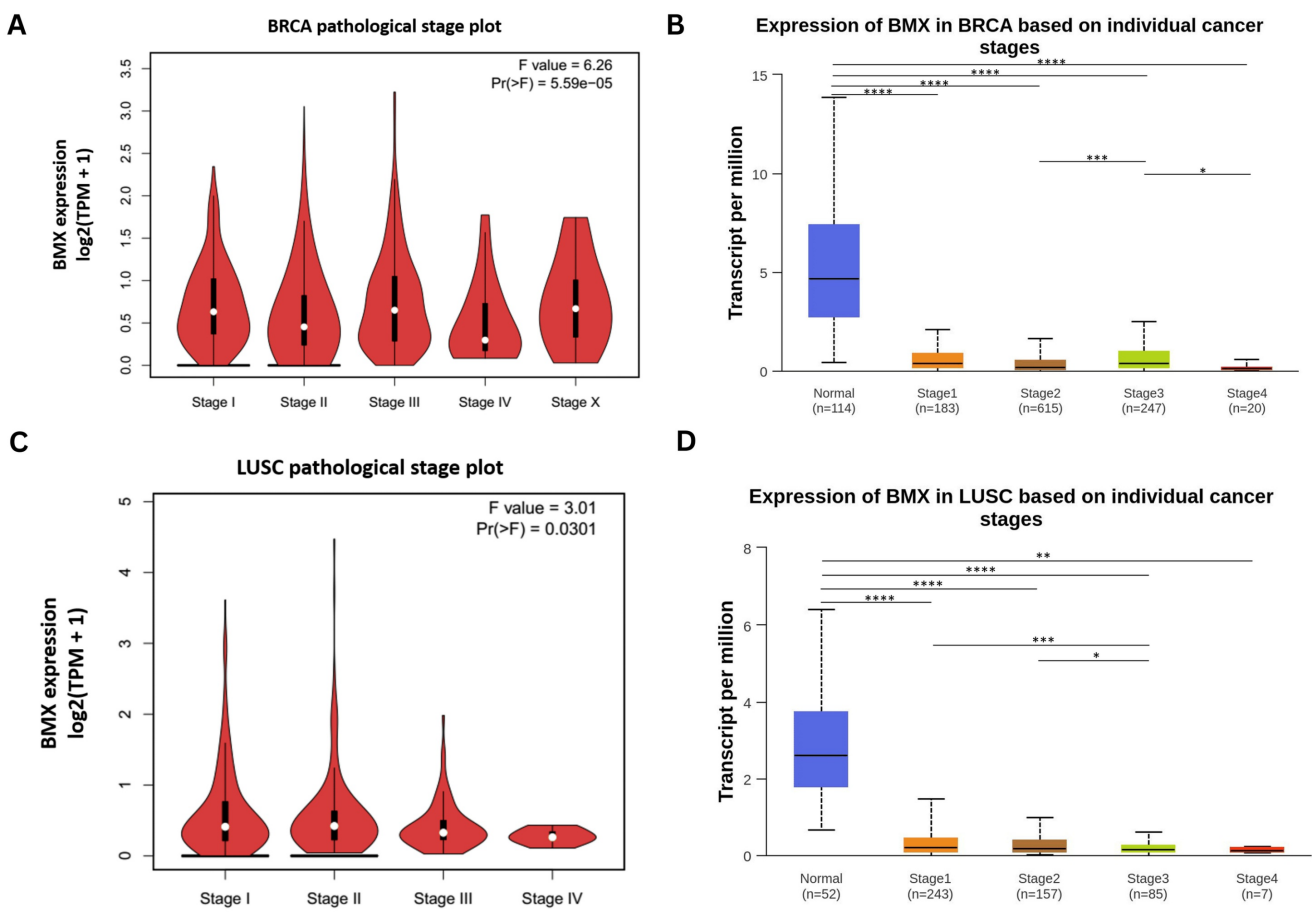
BMX expression analysis in cancer patients according to cancer stage. (A-B) BMX expression in BRCA patients according to cancer stage from GEPIA and UACLAN. (C-D) BMX expression in LUSC patients according to cancer stage from GEPIA and UACLAN. *p < 0.05, **p < 0.01, ***p < 0.001, and ****p<0.0001. BMX: bone marrow kinase; GEPIA: Gene Expression Profiling Interactive Analysis; UALCAN: University of Alabama at Birmingham Cancer Data Analysis; BRCA: breast invasive carcinoma; LUSC: lung squamous cell carcinoma; Portal; Pr(>F): the probability of a greater F-statistic; F value: F-statistic by ANOVA test; p: p-value; TPM: transcript per million.

Our investigation of BMX gene expression patterns across cancer clinical stages utilized data from the GEPIA which individual cancer stages are based on the TCGA clinical annotation [10]. And UACLAN databases which are the cancer stages based on the American Joint Committee on Cancer (AJCC) [11]. ANOVA analysis of the GEPIA data revealed significant differences in BMX expression among the clinical stages of BRCA and LUSC (all p < 0.05; Figure 5A, C), but not in COAD, LUAD, or READ (Appendix D). In comparison, the UACLAN database analysis employed independent t-tests to compare each clinical stage to normal tissue and across stages. This analysis demonstrated that all BRCA stages exhibited significantly lower BMX expression than normal tissue, with stage III being significantly different from stages II and IV (all p < 0.05; Figure 5B). For LUSC, all stages displayed significantly decreased BMX expression compared to normal tissues, with stage III differing significantly from stages I and II (all p < 0.05; Figure 5D). Regarding COAD and READ, all stages showed significant downregulation of BMX relative to normal tissue (all p < 0.0001; Appendix D). In LUAD, stages I, III, and IV were significantly downregulated (all p < 0.05), while stage II did not differ significantly from normal (Appendix D).

BMX expression levels correlated with immunological cell recruitment and infiltration 

**Figure 6:**
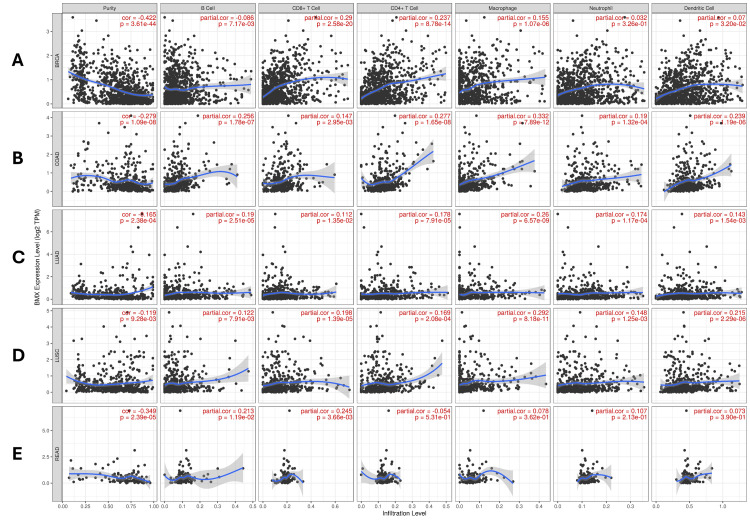
BMX gene expression level correlation with the immune cell recruitment in various cancer types using the TIMER database. (A) BRCA. (B) COAD.  (C) LUAD. (D) LUSC. (E) READ. BMX: bone marrow kinase; TIMER: Tumor Immune Estimation Resource; BRCA: breast invasive carcinoma; COAD: colon adenocarcinoma; LUAD: lung adenocarcinoma; LUSC: lung squamous cell carcinoma; READ: rectum adenocarcinoma; purity: tumor purity; B cells: B lymphocytes; CD8+ T cells: cytotoxic T lymphocytes; CD4+ T cells: T helper cells; cor: correlation; partial cor: partial correlation; p: p-value; TPM: transcript per million.

With the five cancers we previously described by utilizing the TIMER database; we studied the expression of the BMX gene in correlation with the degree of immunological cell infiltration, including; B cells, CD8+ T cells, CD4+ T cells, macrophages, neutrophils, and dendritic cells. In BRCA patients, a statistically significant negative correlation was shown between BMX expression and B cells (Cor = -0.086, p = 0.007). However, there was a positive correlation between BMX expression and CD8+ T cells (Cor = 0.29, p = 2.58E-20), CD4+ T cells (Cor = 0.237, p = 8.78E-14), macrophages (Cor = 0.155, p =1.07E-06), and dendritic cells (Cor = 0.07, p =0.03). Despite this, no statistically significant association was detected between BMX expression and recruitment of neutrophil cells. (Cor = 0.032, p =0.326) (Figure 6A).

Moreover, the abundances of immune cells; B cells (Cor = 0.256, p =1.78E-07), CD8+ T cells (Cor =0.147, p =2.95E-03), CD4+ T cells (Cor = 0.277, p =1.65E-08), macrophages (Cor = 0.332, p =7.89E-12), neutrophil cells (Cor = 0.19, p =1.32E-04), and dendritic cells (Cor = 0.239, p =1.19E-06) were positively correlated with BMX expression in COAD (Figure 6B). In line with this, we also observed that all immune cells, B cells (Cor = 0.19, p =2.51E-05), CD8+ T cells (Cor =0.112, p =0.0135), CD4+ T cells (Cor =0.178, p =7.91E-05), macrophage (Cor = 0.26, p =6.57E-09), neutrophil cell (Cor =0.174, p =1.17E-04), and dendritic cell (Cor = 0.143, p =1.54E-03) in LUAD patients were positively correlated with BMX expression (Figure 6C).

In addition, there was a positive correlation between BMX gene expression and all immune cells abundance; B cells (Cor =0.122, p =7.91E-03), CD8+ T cells (Cor =0.198, p =1.39E-05), CD4+ T cells (Cor =0.169, p =2.08E-04), macrophages (Cor =0.292, p =8.18E-11), neutrophil cells (Cor =0.148, p =1.25E-03), and dendritic cells (Cor = 0.215 p =2.29E-06) in patients with LUSC (Figure 6D).

Finally, in patients with READ; significant positive correlations between BMX expression and B cells (Cor = 0.213, p = 0.0119) and CD8+ T cells (Cor =0.245, p =3.66E-03) were detected, whereas other immune cells were not significantly different (p > 0.05) (Figure 6E). In brief words, a positive correlation of BMX expression levels with the infiltration of all immune cells in five diagnostic cancers was shown, except in BRCA which showed a negative correlation with B cells and no significant correlation with neutrophils.

BMX as a potential biomarker for prognosis

**Figure 7:**
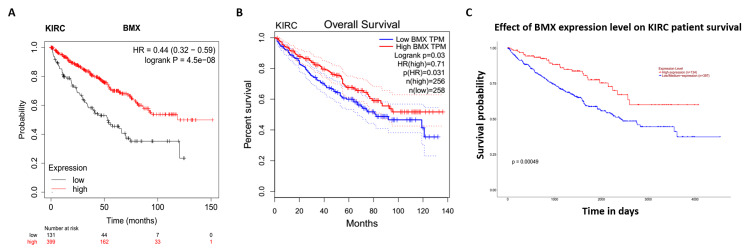
The correlation between the BMX expression level and overall survival outcome of KIRC patients. (A) Kaplan-Meier. (B) GEPIA. (C) UALCAN. BMX: bone marrow kinase; GEPIA: Gene Expression Profiling Interactive Analysis; UALCAN: University of Alabama at Birmingham Cancer Data Analysis Portal; KIRC: kidney renal clear cell carcinoma; HR: hazard ratio; log-rank p: p-value resulting from log-rank test; p: p-value; n: number of samples.

Survival analysis was carried out with data from three databases, Kaplan-Meier plotter, GEPIA, and UALCAN, to determine the correlation between BMX expression and patient overall survival (OS). This study revealed that low expression of BMX was significantly associated with poor prognosis in KIRC patients (Kaplan-Meier plotter: HR= 0.44; p = 4.5e-08; Figure 7A, GEPIA: p = 0.03; Figure 7B, and UALCAN: p = 0.00049; Figure 7C).

**Figure 8:**
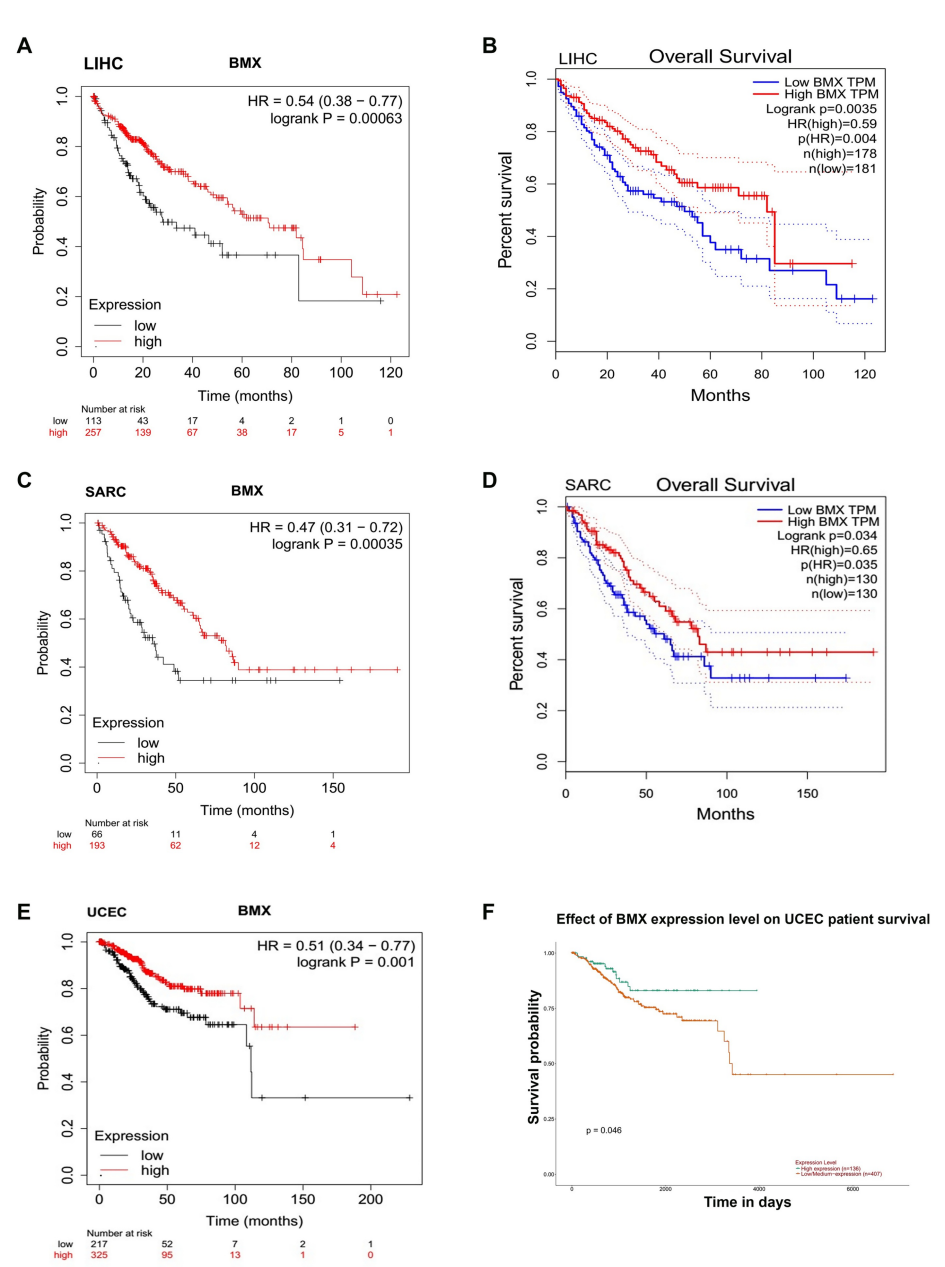
The correlation between the BMX expression level and overall survival outcome of several cancer patients by at least two databases. The relationship between BMX expression and the overall survival outcome of LICH patients according to (A) Kaplan-Meier and (B) GEPIA. SARC patients according to (C) Kaplan-Meier and (D) GEPIA. UCEC patients according to (E) Kaplan-Meier and (F) UALCAN. BMX: bone marrow kinase; GEPIA: Gene Expression Profiling Interactive Analysis; UALCAN: University of Alabama at Birmingham Cancer Data Analysis Portal; LIHC: liver hepatocellular carcinoma SARC: sarcoma; UCEC: uterine corpus endometrial carcinoma; HR: hazard ratio; log-rank p: p-value resulting from log-rank test; p: p-value; n: number of samples.

Besides that, the concordance of findings from at least two databases validated the association between decreased BMX expression levels and unfavorable prognosis in patients diagnosed with LIHC, SARC, and UCEC. (Kaplan-Meier plotter: HR= 0.54; p = 0.00063; Figure 8A and GEPIA: p = 0.0035; Figure 8B), (Kaplan-Meier plotter: HR= 0.47; p = 0.00035; Figure 8C and GEPIA: p = 0.034; Figure 8D), and (Kaplan-Meier plotter: HR= 0.51; p = 0.001; Figure 8E and UALCAN: p = 0.046; Figure 8F) respectively. According to these criteria, there was no significant correlation between OS in patients with high BMX expression and OS in patients with low BMX expression in the remaining cancers (Appendix E and Appendix F). In summary, the low BMX expression levels can be used as an unfavorable prognosis in patients diagnosed with KIRC, LIHC, SARC, and UCEC.

Methylation profile assessment of the BMX DNA promoter

**Figure 9:**
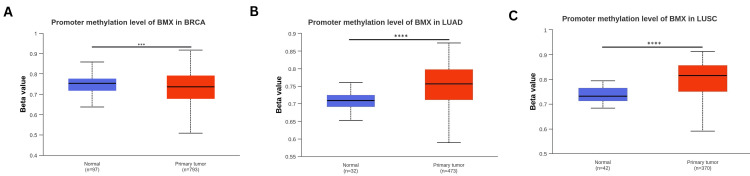
Comparison of the methylation levels of the BMX gene between normal tissues and different tumor tissues using the UALCAN database. (A) BRCA. (B)LUAD. (C) LUSC. ***p < 0.001, ****p<0.0001. BMX: bone marrow kinase; UALCAN: University of Alabama at Birmingham Cancer Data Analysis Portal; BRCA: breast invasive carcinoma; LUAD: lung adenocarcinoma; LUSC: lung squamous cell carcinoma; p: p-value; n: number of samples.

Inserting a methyl group into DNA by a DNA methyltransferase is called DNA methylation, and this heritable (epigenetic) alteration can lead to cancer and other diseases [16]. For this reason, we utilized the UALCAN database in the methylation profile assessment of DNA promoters. A significant downregulation in promoter methylation level of BMX was noticed in BRCA (p < 0.001; Figure 9A), which did not align with its expression. Furthermore, it was significantly upregulated in LUAD (p < 0.0001; Figure 9B) and LUSC (p < 0.0001; Figure 9C), compatible with BMX expression in these cancers. In contrast, there were no significant differences in promoter methylation in COAD and READ tumors in comparison to the normal tissues ( all p > 0.05; Appendix G). The conclusion of the findings showed that LUAD and LUSC had considerably higher methylation levels. But in BRCA, methylation was downregulated.

BMX genetic alteration an in-depth view

**Figure 10:**
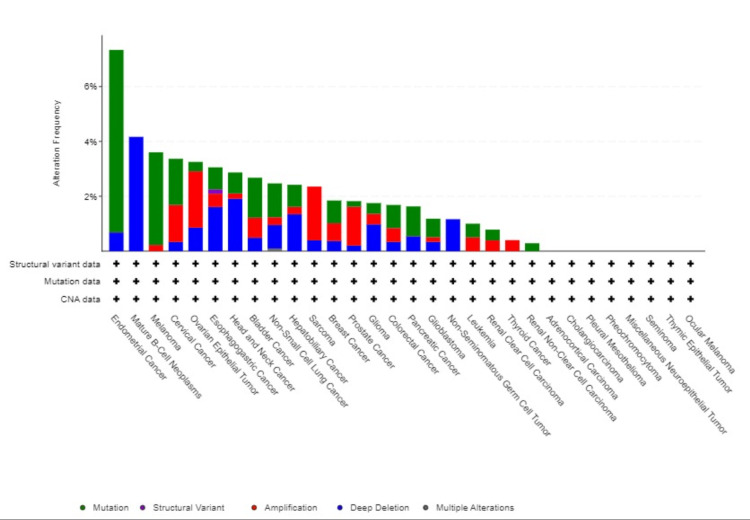
Genetic alteration frequency of BMX in various study cancers using the cBioPortal database. BMX: bone marrow kinase.

We explored genetic variance in BMX across several cancer types using the cBioPortal platform. Using the TCGA dataset, BMX mutation had occurred in 2% of the samples that were tested (10967 samples from 32 studies); the majority of BMX mutations occurred in endometrial cancer with 7.18% (38 patients) mutations and 0.57% (3 patients) deep deletions. Moreover; 0.83% (9 patients) had mutations in BRCA, 0.65% (7 patients) had amplifications, and 0.37% (4 patients) had deep deletions. Similarly, LUSC had mutations in 1.44% (7 patients), amplifications in 0.62% (3 patients), and deep deletions in 1.44% (7 patients). Additionally, 1.06% (6 patients) of LUAD patients had mutations, 0.35% (2 patients) had deep deletions and 0.18% (1 patient) had multiple alterations. BMX expression was not mutated in most samples (Figure 10).

**Figure 11:**
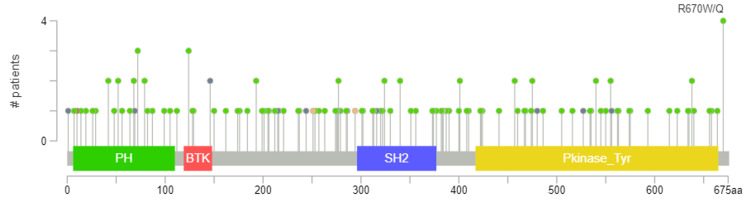
Mutation type and site analysis of BMX among patients with different cancer types using the cBioPortal database. BMX: bone marrow kinase; R670W/Q: amino acid change in which arginine at position 670 changes to either tryptophan or glutamine in the protein sequence; green circles: represent missense mutations; dark gray circles: represent truncating mutations; brown circles: represent inframe mutations; dark orange circles: represent splice mutations; PH: pleckstrin homology domain; BTK: BTK motif; SH2: Src homology-2 domain; Pkinase_Tyr:  protein tyrosine kinase domain.

The majority of mutations in BMX are missense, followed by deep deletions. Moreover, we noticed that the missense variant for BMX was dominant in the first place with position R670W/Q (an amino acid change in which Arginine at position 670 changes to either tryptophan or glutamine in the protein sequence) having the greatest number of alterations among 10,953 samples (Figure 11).

For a more in-depth understanding of BMX genetic alteration's role in carcinogenesis, we inquired about the association of overall outcome between the altered and unaltered BMX groups, and no statistically noteworthy results were shown (p = 0.5) (Appendix H). Ultimately, genetic alteration research showed that just about 2% of the TCGA samples had mutations; the majority of samples did not.

Cross-validation utilizing publicly accessible datasets for BMX expression

**Figure 12:**
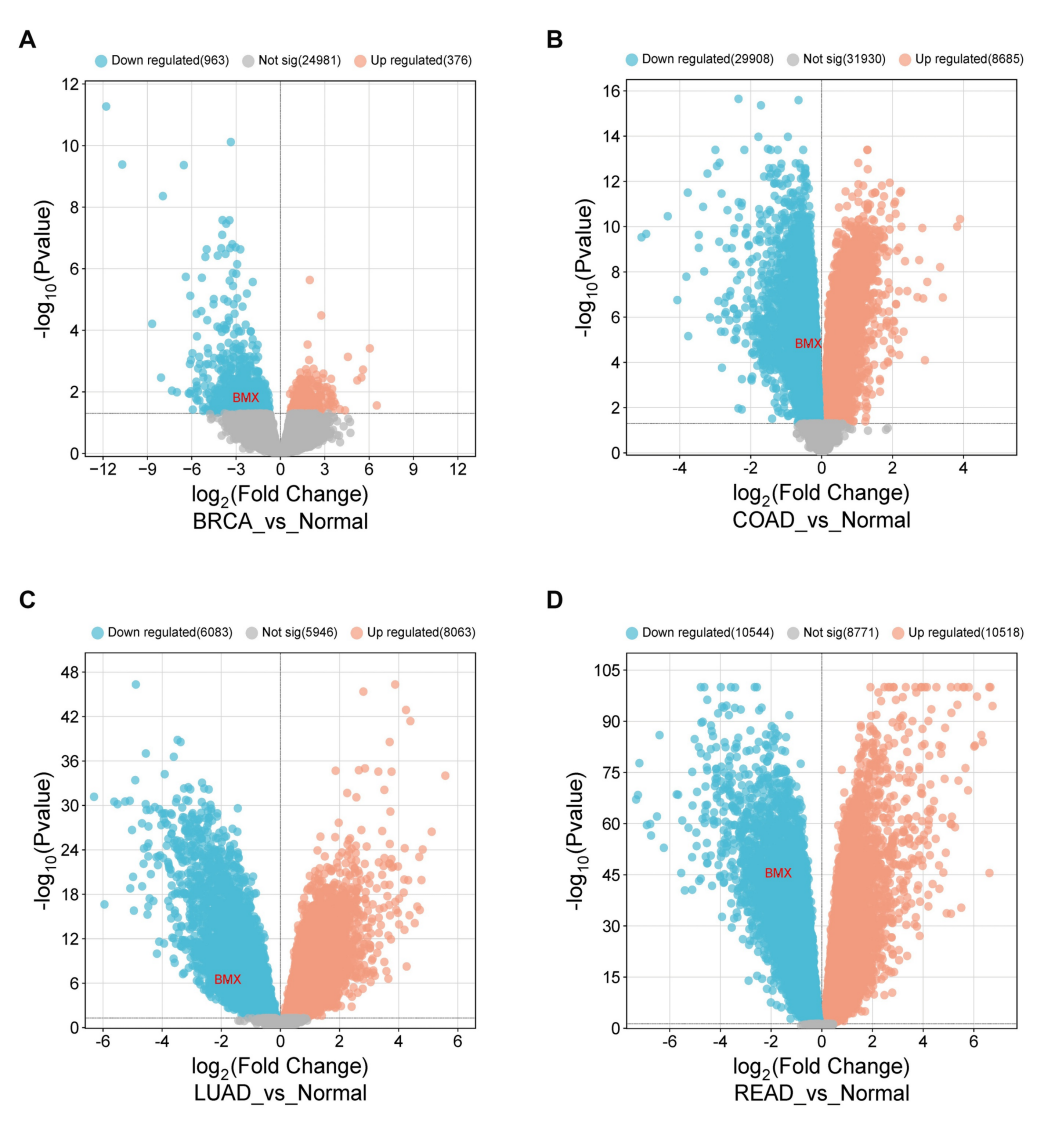
Volcano plots showing differentially expressed genes targeting BMX. (A) BRCA. (B) COAD. (C) LUAD. |Log2FC|>1, adj p-value < 0.05. BMX: bone marrow kinase; BRCA: breast invasive carcinoma; COAD: colon adenocarcinoma; LUAD: lung adenocarcinoma; READ: rectum adenocarcinoma; Log2FC: log2FoldChange; adj p-value: adjusted p-value; Not sig: not significant.

Using publicly available information from the Gene Expression Omnibus (GEO), we verified the expression of BMX across BRCA, COAD, LUAD, LUSC, and READ. The GEO2R tool was successfully employed for assessing the BMX expression across the five diagnostic cancers (|Log2FC| > 1 and adjusted p-value < 0.05). Next, we used the SRplot to create the volcano graphic for differentially expressed genes. The public datasets used were GSE59246 for BRCA; which comprises three samples of normal breast tissue, and 56 samples of invasive breast cancer tissue. A total of 26,320 differentially expressed genes (DEGs) were found in our investigation; 376 of these genes were upregulated and 963 were downregulated. BMX was significantly downregulated (logFC= -2.3238, adj.P.Val= 0.0168) (Figure 12A). Similarly, we used the GSE84984 dataset for COAD validation, in which seven tumor samples and six normal samples were used; the analysis revealed that 70,523 DEGs, 29908 downregulated, and 8685 upregulated, and BMX within the downregulated genes (logFC= -0.367636, adj.P.Val= 1.63E-05) (Figure 12B). Furthermore, GSE115002 was used to analyze the mRNA expression of every possible gene in 52 paired LUAD and normal tissues that were adjacent via genome-wide microarray analysis. The analysis revealed 20,092 DEGs; 6083 were downregulated, 8063 were upregulated, and BMX was downregulated (logFC= -1.7746697, adj.P.Val= 4.36E-07) (Figure 12C). Last but not least, the GSE87211 Dataset of READ gene expression microarray of 230 cancer samples and matched 160 mucosa control has been done. The analysis revealed 29,833 DEGs; 10544 were downregulated, 10518 were upregulated, and BMX was one of the downregulated genes (logFC= -1.7098967, adj.P.Val= 6.66E-46) (Figure 12D). In contrast, there was not a statistically significant distinction in BMX expression in LUSC, but it persisted downregulated (Appendix I)

## Discussion

BMX, a bone marrow kinase, pertains to the nonreceptor tyrosine kinase family alongside gene synonyms ETK and PSCTK3. Situated cryogenically at Xp22.2 on the forward strand, this gene exhibits five transcripts, three of which are responsible for encoding proteins as documented in the Ensembl database [17]. Considering the lack of earlier direct research into the relationship between the BMX gene and its role in cancer, our team chose to explore this promising study subject matter; this study was committed to examining the possible function of BMX as a biomarker for both the diagnosis and prognosis of various cancer types. Hence, BMX was systematically characterized across 33 TCGA tumor types through the analysis of gene expression, which was further validated using various datasets to confirm our gene expression findings. Moreover, to gain a comprehensive understanding of the role of the BMX gene in cancer, we delved into clinical parameters, immune infiltration, methylation profiles, and genetic alterations.

Using TCGA samples, this analysis revealed that BMX gene expression was significantly downregulated in several cancer types, including BRCA, COAD, LUAD, LUSC, and READ, compared with normal samples in the TIMER, GEPIA, and UALCAN databases. The consistent downregulation of BMX expression observed across multiple cancer datasets underscores the potential utility of this gene as a valuable diagnostic biomarker for these cancers. According to earlier research, BMX plays a critical role in several biological processes, such as inflammation, cardiovascular disease, cancer, particularly glioblastoma cancer stem cells, and solid tumors such as breast and prostate cancer [6,7]. However, few previous studies have investigated the direct diagnostic role of BMX in cancer; one study reported that BMX expression was greater in colon, breast, and prostate tumors than in normal tissue [1], which contradicts our findings. These contrary results may be due to the different samples used to assess BMX expression; in this study, we used RNA sequences from the TCGA dataset, whereas the previous study utilized cancer cell lines [1]. Gene expression levels vary between cell lines and their original tissues due to differences in regulatory mechanisms, epigenetic modifications [18], and the influence of the cellular microenvironment [19]. 

A detailed analysis revealed consistent downregulation of BMX expression across various patients' clinical parameters, including age, gender, race, and clinical stage, in the five cancer types compared to the normal tissues; notably, stage III was significantly varied from stage II, and stage IV in BRCA, and LUSC with stage III significantly varied from stage I and stage II which agreed with the analysis performed by GEPIA. These results emphasize that the BMX gene is involved in cancer progression, and could serve as a biomarker for staging, a therapeutic target, and a prognostic indicator in BRCA and LUSC. To our knowledge, this is the first study to demonstrate the correlation between BMX expression and clinicopathological characteristics. These findings warrant further investigation to understand the precise mechanisms behind this association. These findings can contribute to the efforts of previous studies [20,21] toward these cancers by improving diagnosis, treatment strategies, and overall patient outcomes.

To gain a thorough understanding of the significance of BMX as a therapy within the tumor microenvironment, we used the TIMER database to examine its correlation with immune cell infiltration, which includes B cells, CD8+ T cells, CD4+ T cells, neutrophils, macrophages, and dendritic cells across BRCA, COAD, LUAD, and LUSC; a positive correlation with the infiltration of all immune cells in these cancers was shown, except in BRCA which showed a negative correlation with B cells and no significant correlation with neutrophils. However, in READ, BMX expression was significantly positively correlated with only B cells and CD8+ T cells. In response to TNFα, IL-1β, and TLR agonists, BMX controls the release of proinflammatory cytokines and important signaling pathways such as MAP kinase and NFκB, which affect inflammation [6]. According to a previous study, BMX is one of the 11 hub genes that may be targeted for therapy. The associations between these genes and NK cells, plasma cells, neutrophils, and macrophages may influence the carcinogenesis of specific malignancies [22]. The study has been mentioned and this study built upon each other to explore the potential of BMX as a target in the fight against cancer. However, these findings are preliminary and require further experimental and clinical validation to confirm the role of BMX in cancer therapy and its potential as a therapeutic target.

Moreover, this study displays that BMX is a prognostic biomarker by using at least two databases from Kaplan-Meier, GEPIA, and UALCAN databases, where we found that low expression of BMX significantly correlated with poor prognosis in KIRC, LIHC, SARC, and UCEC patients. In contrast, an earlier study documented that elevated BMX expression in aggressive cancers such as prostate, breast, and colon cancers is linked to heightened apoptosis resistance and poorer patient survival outcomes [1], indicating that the role of BMX in cancer may be complex and context-dependent. Therefore, further investigations are required to elucidate the underlying mechanisms and context-dependent functions of BMX in various cancer types.

Similarly, a promoter methylation profile review was performed to analyze the promoter methylation levels of BMX. The results revealed that the methylation levels were significantly increased in LUAD and LUSC. However, methylation was downregulated in BRCA. One method of altering gene expression is through limiting gene transcription, which is possible since RNA polymerases are unable to identify methylated DNA [16]. Importantly, our findings of significantly low BMX expression in LUAD and LUSC, along with their methylation profiles suggest that the low expression of BMX across these cancers is due to epigenetic factors. This finding was further confirmed by alteration analysis, which revealed that a small percentage of LUSC and LUAD patients had genetic alterations in BMX. On the other hand, the distinct pattern of BMX downregulation observed in BRCA, where methylation was downregulated with minimal genetic alterations, emphasizes the complexity of BMX regulatory mechanisms. This underscores the need for further investigation to fully elucidate the specific mechanisms driving the observed BMX downregulation in BRCA.

Limitations of the study 

First, while this study provides useful insights, converting in silico predictions to real-world clinical applications requires additional in vivo or in vitro confirmation. Second, the study is greatly influenced by the quality and relevance of the data sources employed; biases or restrictions in the datasets might produce misleading conclusions. Finally, the study concentrated on BMX expression levels and their relationship with clinical outcomes in various malignancies, ignoring potential regulatory mechanisms and connections with other molecular pathways. Despite these constraints, the utilization of TCGA data is a crucial initial phase toward acknowledging the future direction of BMX.

## Conclusions

This study revealed BMX as a diagnostic biomarker in BRCA, COAD, LUAD, LUSC, and READ and a prognostic biomarker in KIRC, LIHC, SARC, and UCEC. Furthermore, epigenetic variables may have a greater impact on BMX expression levels especially in LUAD and LUSC. This study also emphasized the role of BMX in immune cell infiltration, such as B cells, CD4+ T cells, CD8+ T cells, neutrophils, macrophages, and dendritic cells in certain cancers. The BMX expression level highlights the prognostic value and potential therapeutic potential of BMX.

## Figures and Tables

**Figure 13:**
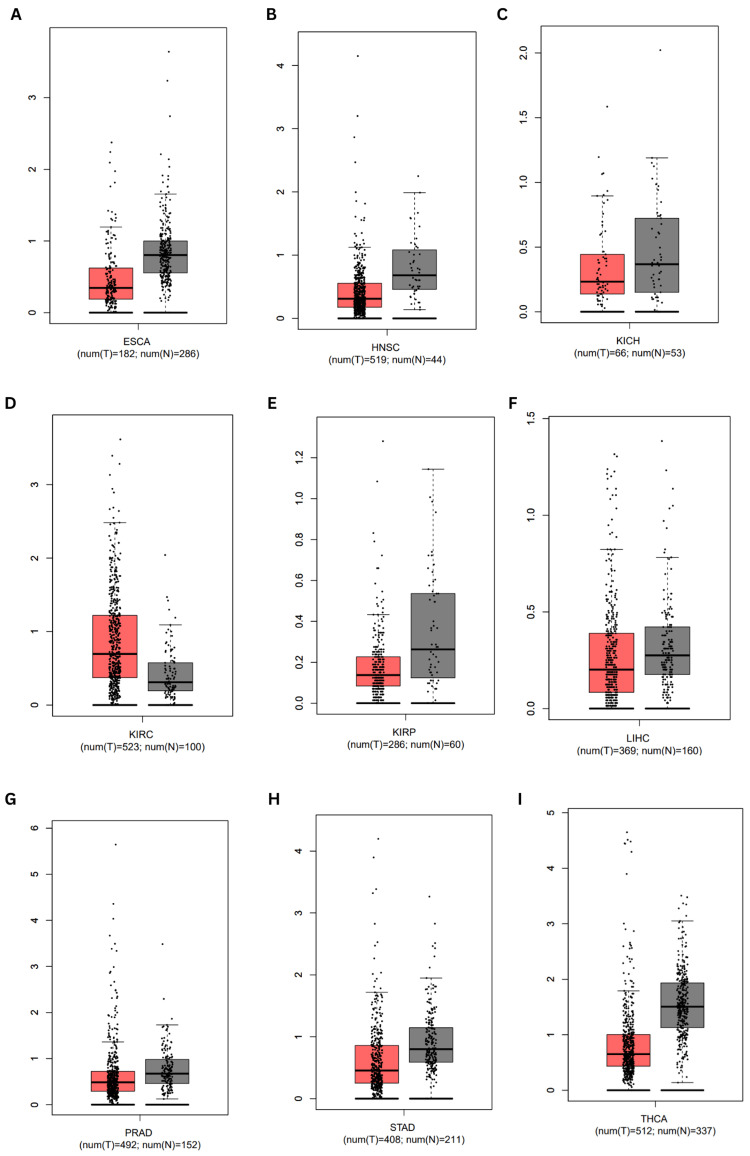
BMX expression among non-significant various types of cancer according to the GEPIA database. (A) ESCA. (B) HNSC. (C) KICH. (D) KIRC. (E) KIRP. (F) LICH. (G) PRAD. (H) STAD. (I) THCA. BMX: bone marrow kinase; GEPIA: Gene Expression Profiling Interactive Analysis; ESCA: esophageal carcinoma; HNSC: head and neck squamous cell carcinoma; KICH: kidney chromophobe; KIRC: kidney renal clear cell carcinoma; KIRP: kidney renal papillary cell carcinoma; LIHC: liver hepatocellular carcinoma; PRAD: prostate adenocarcinoma; STAD: stomach adenocarcinoma; THCA: thyroid carcinoma; num(T): the number of tumor samples; num(N): the number of normal samples; red color: tumor samples; gray color: normal samples; Y axis: level of BMX expression in transcripts per million.

**Figure 14:**
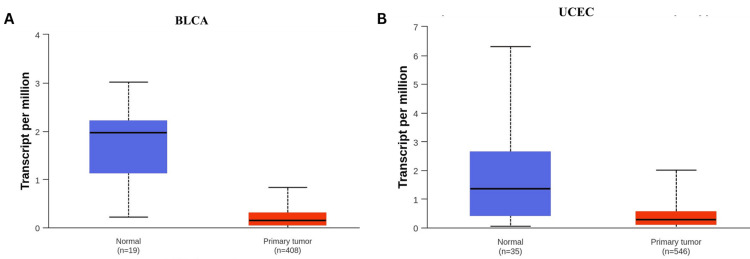
BMX expression among non-significant various types of cancer according to the UACLAN database. (A) BMX expression in BLCA. (B) BMX expression in UCEC. BMX: bone marrow kinase; UALCAN: University of Alabama at Birmingham Cancer Data Analysis Portal; p: p-value; BLCA: bladder urothelial carcinoma; UCEC: uterine corpus endometrial carcinoma.

**Figure 15:**
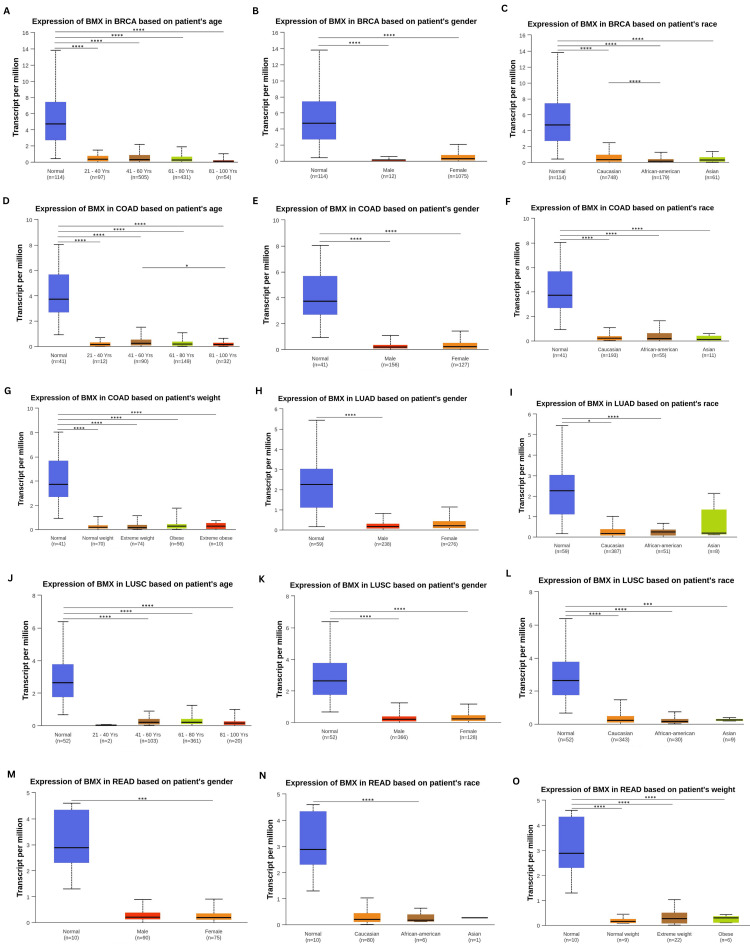
BMX expression analysis in cancer patients according to certain clinical parameters (age, gender, race, and weight) according to the UALCAN database. (A-C) BMX expression in BRCA patients according to age, gender, and race. (D-G) expression of BMX in COAD patients based on age, gender, race, and weight. (H-I) BMX expression in LUAD patients according to gender and race. (J-L) BMX expression in LUSC patients according to age, gender, and race. (M-O) expression of BMX in READ patients based on gender, race, and weight.*p < 0.05, ***p < 0.001, and ****p<0.0001. BMX: bone marrow kinase; UALCAN: University of Alabama at Birmingham Cancer Data Analysis Portal; p: p-value; BRCA: breast invasive carcinoma; COAD: colon adenocarcinoma; LUSC: lung squamous cell carcinoma; READ: rectum adenocarcinoma.

**Figure 16:**
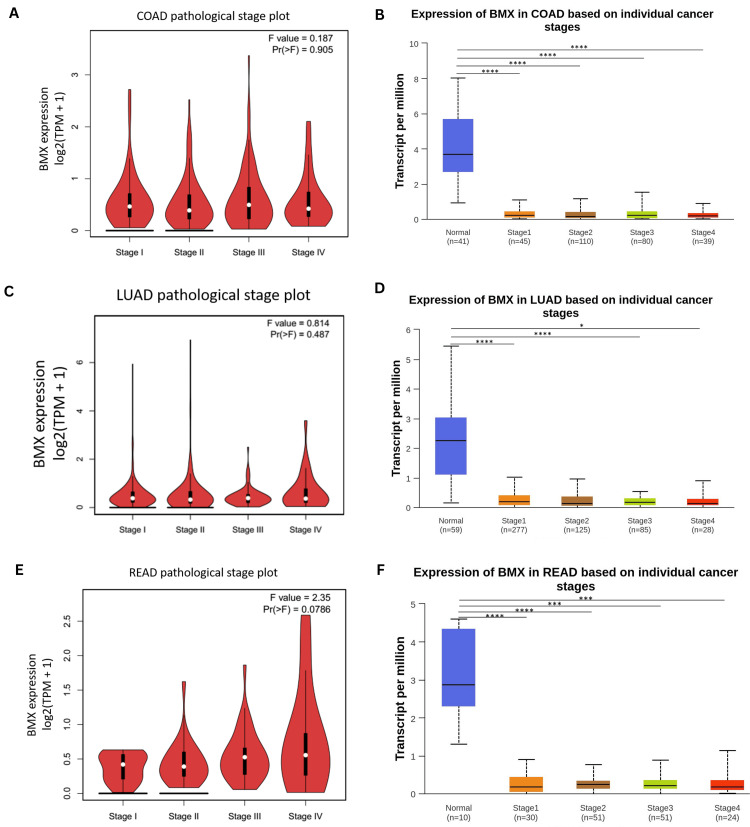
BMX expression analysis in cancer patients according to cancer stages. (A-B) BMX expression in COAD patients according to cancer stage according to GEPIA and UACLAN. (C-D) BMX expression in LUAD patients according to cancer stage from GEPIA and UACLAN. (E-F) BMX expression in READ patients according to cancer stage from GEPIA and UACLAN. *p < 0.05, ***p < 0.001, and ****p<0.0001. BMX: bone marrow kinase; GEPIA: Gene Expression Profiling Interactive Analysis; UALCAN: University of Alabama at Birmingham Cancer Data Analysis Portal; p: p-value; COAD: colon adenocarcinoma; LUAD: lung adenocarcinoma; READ: rectum adenocarcinoma; TPM: Transcript per million.

**Figure 17:**
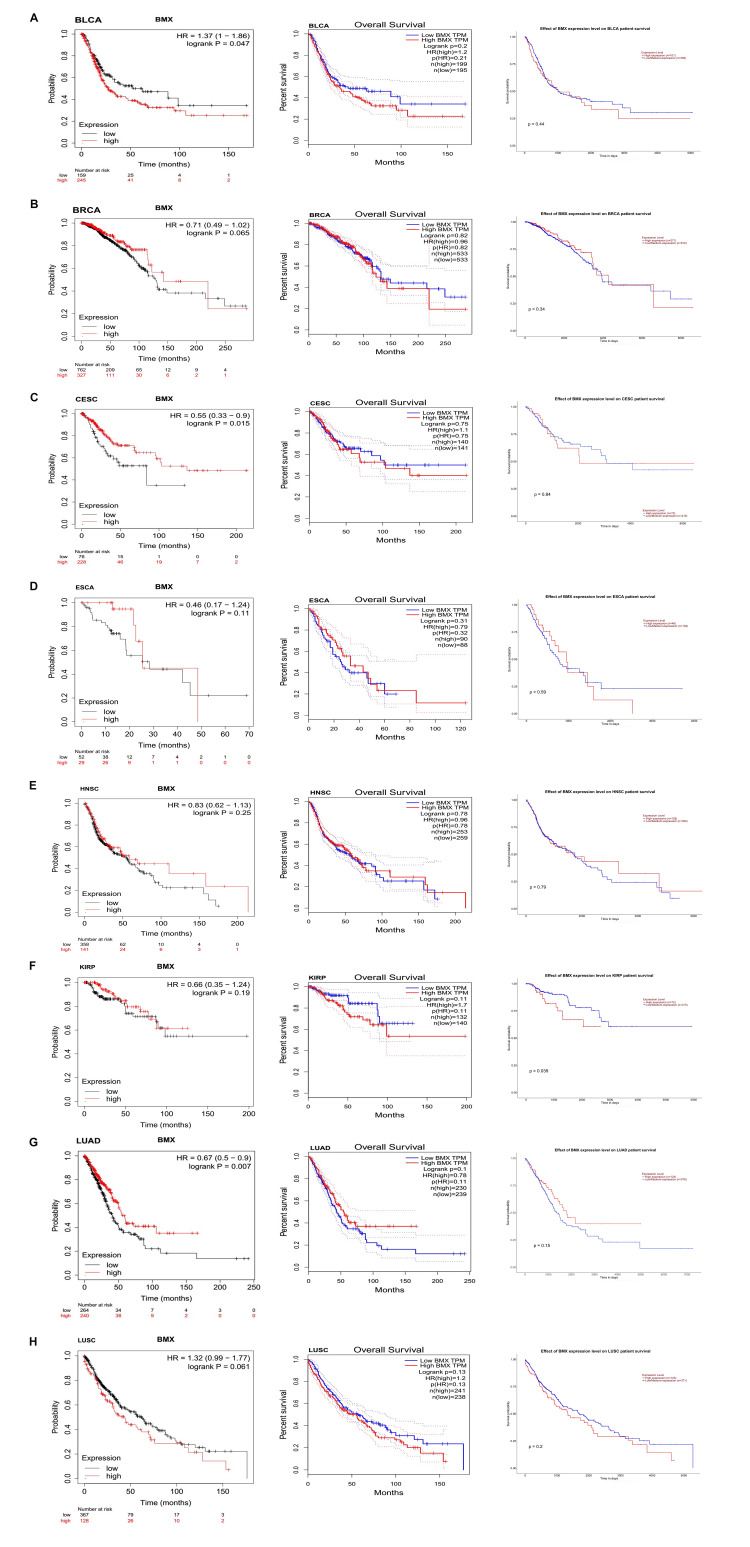
The correlation between the BMX expression level and the survival outcome of patients using Kaplan-Meier plotter, GEPIA, and UALCAN databases. The relationship between BMX expression and the overall survival of patients with (A) BLCA, (B)BRCA, (C) CESC, (D) ESCA, (E) HNSC, (F) KIRP, (G) LUAD, and (H) LUSC from Kaplan-Meier Plotter, GEPIA, and UALCAN databases respectively. BMX: bone marrow kinase; GEPIA: Gene Expression Profiling Interactive Analysis; UALCAN: University of Alabama at Birmingham Cancer Data Analysis Portal; BLCA: bladder urothelial carcinoma; BRCA: breast invasive carcinoma; CESC: cervical squamous cell carcinoma and endocervical adenocarcinoma; ESCA: esophageal carcinoma; HNSC: head and neck squamous cell carcinoma; KIRP: kidney renal papillary cell carcinoma; LUAD: lung adenocarcinoma; LUSC: lung squamous cell carcinoma; HR: hazard ratio; log-rank p: p-value resulting from log-rank test; p: p-value; n: number of samples; TPM: Transcripts per million.

**Figure 18:**
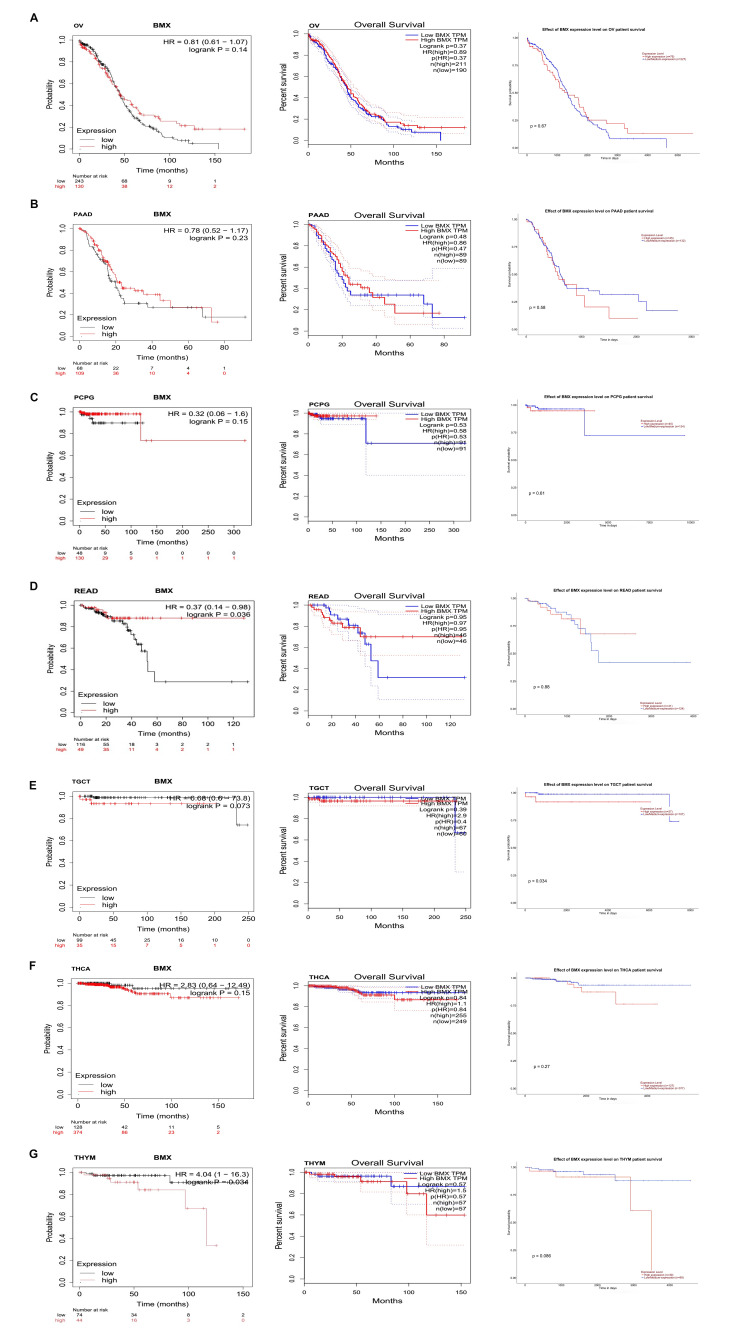
The correlation between the BMX expression level and the survival outcome of patients using Kaplan-Meier plotter, GEPIA, and UALCAN databases. The relationship between BMX expression and the overall survival of patients with (A) OV, (B) PAAD, (C) PCPG, (D) READ, (E) TGCT, (F) THCA, and (G) THYM from Kaplan-Meier Plotter, GEPIA, and UACLAN  respectively. BMX: bone marrow kinase; GEPIA: Gene Expression Profiling Interactive Analysis; UALCAN: University of Alabama at Birmingham Cancer Data Analysis Portal; OV: ovarian serous cystadenocarcinoma; PAAD: pancreatic adenocarcinoma; PCPG: pheochromocytoma and paraganglioma; READ: rectum adenocarcinoma; TGCT: testicular germ cell tumors; THCA: thyroid carcinoma; THYM: thymoma; HR: hazard ratio; log-rank p: p-value resulting from log-rank test; p: p-value; n: number of samples; TPM: Transcripts per million.

**Figure 19:**
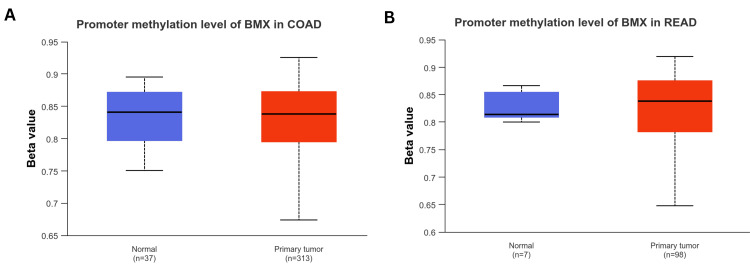
Methylation levels of BMX gene between normal tissues and non-significant different tumor tissues, conducted using the UALCAN database. (A)COAD. (B) READ. BMX: bone marrow kinase; UALCAN: University of Alabama at Birmingham Cancer Data Analysis Portal; COAD: colon adenocarcinoma; READ: rectum adenocarcinoma; p: p-value; n: number of samples.

**Figure 20:**
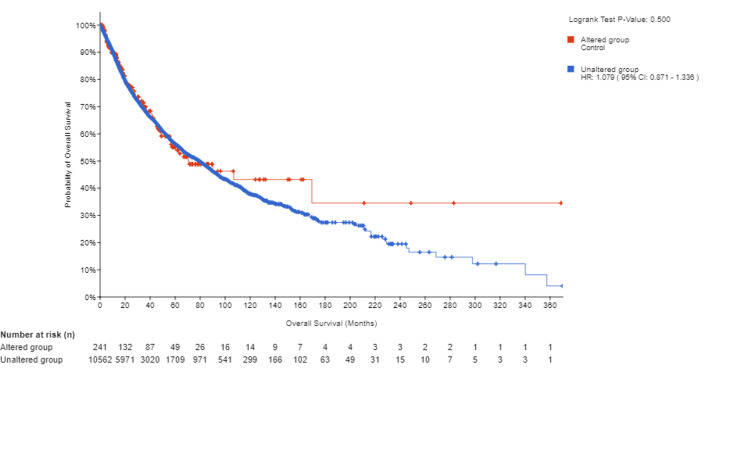
Correlation of overall survival outcomes between the altered BMX gene and unaltered groups using the cBioPortal database. BMX: bone marrow kinase; log-rank P: p-value resulting from log-rank test.

**Figure 21:**
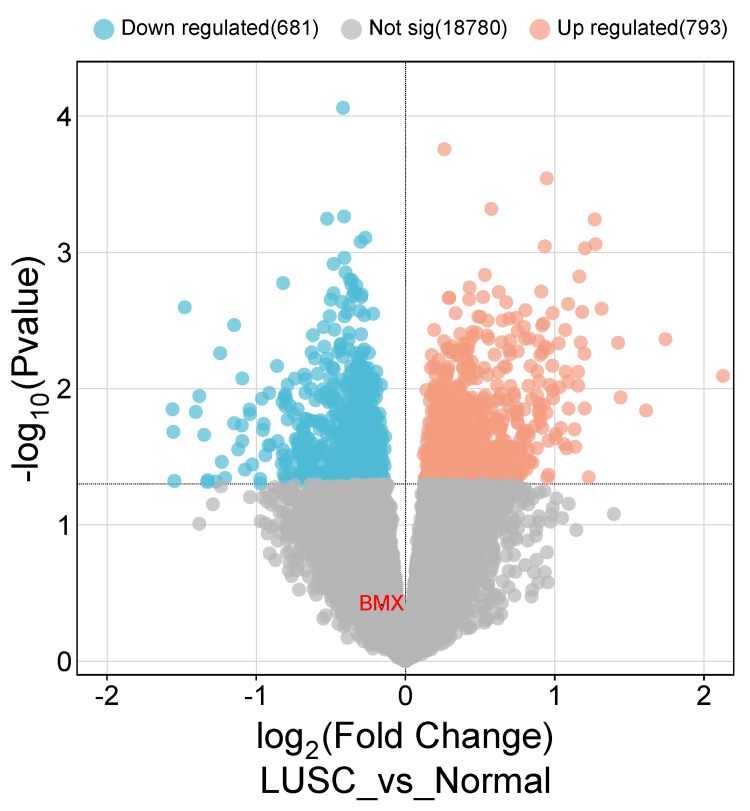
Volcano plots illustrate differential gene expression, targeting BMX in LUSC patients, using the GSE134381 dataset. |Log2FC|>1, adj p-value < 0.05. BMX: bone marrow kinase; LUSC: lung squamous cell carcinoma; Log2FC: log2 Fold Change; adj p-value: adjusted p-value; Not sig: not significant.

## Appendices

Appendix A

Appendix B

Appendix C

Appendix D

Appendix E

Appendix F

Appendix G

Appendix H

Appendix I
